# Health Related Quality of Life among Children with Sickle Cell Anaemia in Northwestern Tanzania

**DOI:** 10.4236/ojbd.2022.122002

**Published:** 2022-05-23

**Authors:** Zivonishe Mwazyunga, Emmanuela E. Ambrose, Neema Kayange, Respicious Bakalemwa, Benson Kidenya, Luke R. Smart, Adolfine Hokororo

**Affiliations:** 1 Department of Paediatrics, Catholic University of Health & Allied Sciences and Bugando Medical Centre, Mwanza, Tanzania; 2 Division of Haematology, Cincinnati Children’s Hospital, Cincinnati, OH, USA

**Keywords:** Sickle Cell Anaemia, Health, Quality of Life, Children, Tanzania

## Abstract

**Background:**

Sickle cell anaemia (SCA) is a serious, multisystem, genetic disorder affecting millions of children worldwide. The disease causes numerous complications that interfere with the health-related quality of life (HRQoL) of these children including an impact on educational, physical and psychosocial development. Few studies have described the clinical spectrum and quality of life of children with SCA living in a low-resource area.

**Objectives:**

This study aimed to determine the clinical spectrum and HRQoL among children living with sickle cell anaemia (SCA) in northwest Tanzania.

**Methods:**

This hospital-based cross-sectional study took place at Tertiary and teaching hospital, Bugando Medical Centre, Mwanza Tanzania. The study enrolled children ages 2 – 12 years old with SCA attending the Bugando Medical Centre sickle cell clinic. Health related quality of life was measured using the Pediatric Quality of Life, Brief Generic Core Scale after translating from English into a Swahili version. Important SCA complications were assessed using a structured questionnaire.

**Results:**

From October 2016 to March 2017, 204 children were enrolled. Participants presented at a median age of 6 years [IQR 4 – 9]. Among children with SCA the most common clinical signs at the time of enrolment were pale in 69.6% (142/204), jaundice in 65.9% (134/204), oxygen saturation < 90% in 25% (51/204) and splenomegaly in 19% (39/204). Severe anaemia was observed in 30.9% (63/204). A majority reported vaso-occlusive crisis (166/204, 81.4%), and very few had experienced a prior stroke (5/204, 2.5%). Using a modified Likert scale, a total of 41/204 (20.1%) children had poor HRQoL indicated by low scores on PedsQL^™^ and 163/204 (79.9%) children had high scores, indicating good HRQoL. On multivariate analysis, age ≥ 5 years (p-value < 0.001), haemoglobin < 7 g/dl (p-value = 0.001) and >3 hospitalizations per year (p-value = 0.008) were associated with poor HRQoL.

**Conclusion:**

SCA complications, negatively impact the HRQoL of children living with the disease. Severe anaemia, older age and frequent hospitalizations were highly associated with poor HRQoL. Comprehensive management is needed beginning at diagnosis to identify these children early and provide them with adequate support.

## 1. Introduction

Sickle cell anemia (SCA) is a genetic disorder with many physical manifestations. The clinical course is characterized by periods of quiescence and exacerbation, but hallmarks of the disease include chronic haemolytic anaemia, infection, and vaso occlusive episodes that cause acute pain, acute chest syndrome, and damage to cerebral vasculature that increases the risk of stroke [1] [2] [3].

The physical manifestations of SCA have a significant impact on quality of life (QoL). QoL is a person’s ability to perform the ordinary tasks of daily living. It moves beyond direct manifestations of illness to the patient’s personally experienced morbidity. Assessment of QoL is an important aspect of chronic disease management [2]. Children with SCA may have restrictions placed on their physical activities such as performing household chores, play, or self-care [3]. Exacerbations and hospitalizations may impede school attendance and lead to compromised physical, psychological and cognitive function, particularly attention and learning problems in children [4].

SCA is common in sub-Saharan Africa, but its complications and effect on QoL in the African setting are poorly understood. Physical manifestations that affect QoL in SCA are particularly common in sub-Saharan Africa. Northwest Tanzania has a particularly high prevalence of SCA and a high burden of associated complications [5] [6] [7] [8]. Children in such settings receive a basic package of preventive measures (penicillin, mosquito bed nets, folic acid), but the bulk of the care is focused on symptomatic management of disease exacerbations and complications. While several studies have demonstrated the effect of SCA on QoL in children living with SCA in the USA, little is understood about SCA and QoL in sub-Saharan Africa [9] [10] [11]. Psychosocial needs are rarely assessed or addressed in daily practice.

Therefore, we set out to determine the clinical spectrum and Health Related Quality of Life (HRQoL) among children living with SCA in northwest Tanzania so that we could better understand the usefulness of assessing HRQoL in daily practice. Our secondary objective was to determine factors associated with poor HRQoL so that targeted therapies could be provided to prevent worsening of HRQoL. With a proper understanding of the severity of the problem and its associated factors, appropriate services such as cognitive-behavioural therapy, occupational therapy, or physical therapy, could be arranged for patients earlier in their disease course.

## 2. Material and Methods

### 2.1. Study Area and Recruitment

**Figure 1.**
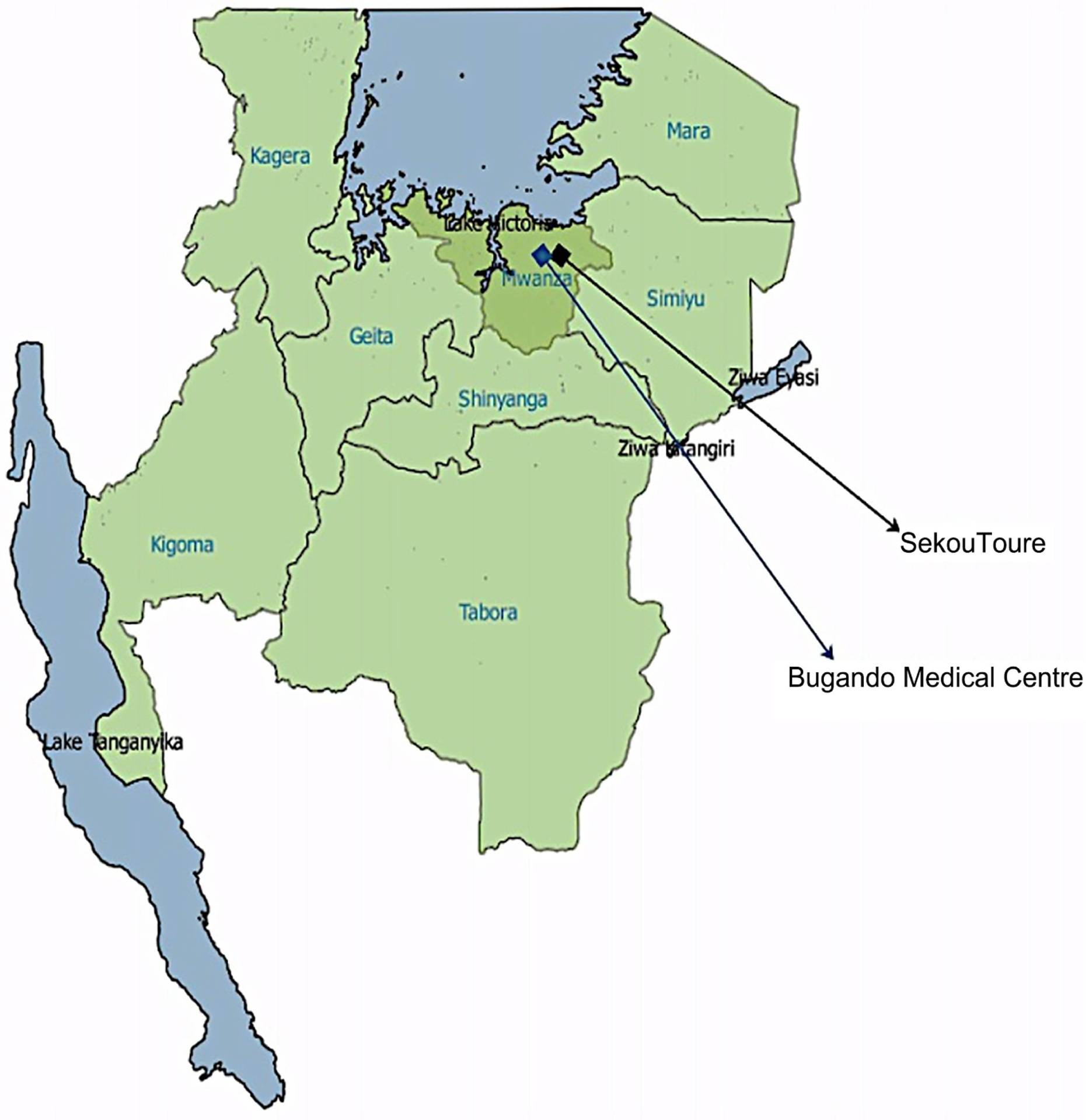
The map of Tanzania north western regions served by bugando medical centre driven from Tanzania national bureau of statistics regions shapefile (2012) http://www.nbs.go.tz/nbs/takwimu/references/GIS_Maps.zip region selection version QGIS 2.12.0 http://www.qgis.org/en/site/forusers/download.html.

This was a hospital-based cross-sectional study that was conducted among children with SCA attending the outpatient paediatric clinic at Bugando Medical Centre (BMC) in northwest Tanzania. BMC is a zonal referral hospital as well as a consultant and tertiary specialist teaching hospital responsible for care of all the Lake and Western Zones of the United Republic of Tanzania. The catchment area includes nine regions with a population of approximately 16.2 million people (see Figure 1). BMC has a paediatric sickle cell clinic with >500 children enrolled. The clinic runs twice a week, and about 50 patients are seen on a weekly basis. Most of the children initially report to the clinic after development of complications such as dactylitis, recurrent fever, or vaso-occlusive crisis.

Children with SCA, genotype HbSS, from 2 to 12 years old attending SCA clinic at BMC were eligible for the study. We excluded all hospitalised children with SCA. After written informed consent was obtained from the parents or guardians, demographic and clinical information were collected using a standardized questionnaire. A complete physical examination of each child was performed, including measurements of weight and height using a standardized WHO chart, and BMI was calculated. Nutritional status Z-score was determined for each child.

### 2.2. Health Related Quality of Life

The Pediatric Quality of Life (PedsQL^™^) Brief Generic Core Scale was used (after translating from English to Swahili) to measure HRQoL for each child [12]. For children 2 – 4 years old, the parental proxy questionnaire was used, while for children 5 – 12 years old a self-report questionnaire was used [13]. The PedsQL^™^ Brief Generic Core Scale is composed of 23 questions about problems with different aspects of daily life in 4 different domains: physical functioning, social functioning, emotional functioning, and school functioning. The questions within each domain are answered using a Likert scale from 0 (never a problem) to 4 (almost always a problem), so that higher scores suggest a lower HRQoL.

For ease of statistical evaluation and interpretation, the scores were reversed and transformed to a 0 – 100 scale so that higher scores indicate better HRQoL. The initial 0 – 4 Likert scale values were reversed and converted to 0 – 100 as follows: 0 = 100, 1 = 75, 2 = 50, 3 = 25, 4 = 0. A final score for each domain was calculated by dividing the sum of the scores for a domain by the number of questions within that domain. A total score was calculated in the same fashion by taking the sum of all scores in the questionnaire by the number of questions in the questionnaire. Using this method, the established cut off below which a child is considered to have poor quality of life is 69.7 for children 5 – 12 years old who are self-reporting and below 65.4 for parental proxy reporting among children of 2 – 4 years [13].

### 2.3. Laboratory Methods

Peripheral venous blood was collected in ethylene diamine tetra-acetic acid (EDTA) tubes at enrolment from all eligible children. A complete blood count (CBC) including haemoglobin concentration, mean corpuscular volume (MCV), mean corpuscular haemoglobin concentration (MCHC), red blood cell count, reticulocyte count, leucocyte count and platelet count were determined using a Mindray BC 3200 Haematology Analyser (Mindray, Nanshan, Shenzhen, China).

### 2.4. Data Analysis

Data was entered into Microsoft Excel (Microsoft, Redmond, Washington, U.S.A.) and was exported to Stata version 13 (StataCorp LLC, College Station, Texas, U.S.A.) for analysis. Continuous data were summarized using means and standard deviations (SD) or medians and interquartile ranges [IQR] while categorical data were summarized using proportions. Anaemia was categorized according to the World Health Organization as severe anaemia (<7.0 g/dL), moderate anaemia (7.0 – 9.9 g/dL), or mild anaemia (10.0 – 10.9) [14]. HRQoL was initially recorded as a continuous variable and then categorized as either poor QoL or good QoL. To determine factors associated with poor HRQoL, univariate logistic regression was performed. Factors with a p-value less than 0.1 in univariate logistic regression model were then subjected to multivariate logistic regression analysis. Factors with a p-value of less than 0.05 were considered to be independently associated with HRQoL.

## 3. Results

### 3.1. Enrolment Procedure

**Figure 2.**
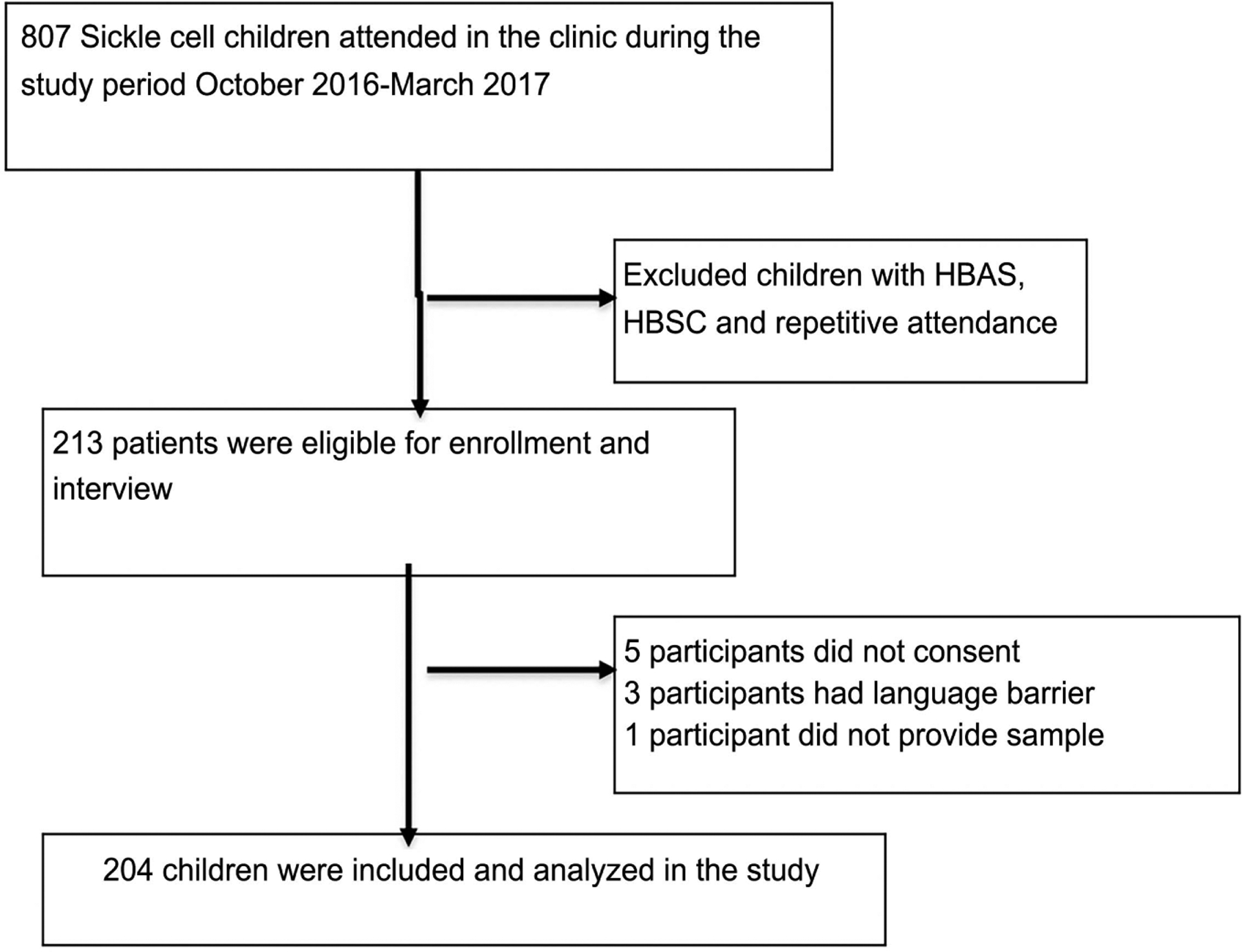
Enrollment procedure of children with Sickle cell children attended in the clinic during the study period October 2016-march 2017.

From October 2016 to March 2017, there were 807 outpatient visits in the BMC sickle cell clinic. After exclusion of repeat visits and children with HbAS, 213 children with SCA were eligible for the study. A total of 204 consented and were enrolled into the study (see Figure 2).

Their median age was 6 [4 – 9] years and males comprised 61.3% (125/204). The median age at which children reported having initially presented to the sickle cell clinic with signs and symptoms of SCA was 12 months [IQR 6 – 48]. Most of the children (90.2%, 184/204) were brought by their parents to the sickle cell clinic (Table 1).

### 3.2. Clinical Findings

Among children with SCA the most common clinical signs at the time of enrolment were pale in 69.2% (141/204), jaundice in 65.7% (134/204), oxygen saturation < 90% in 25% (51/204) and splenomegaly in 19.1% (39/204). Anaemia severity was moderate in 64.2% (131/204), and severe in 30.9% (63/204) (see Table 2).

**Figure 3.**
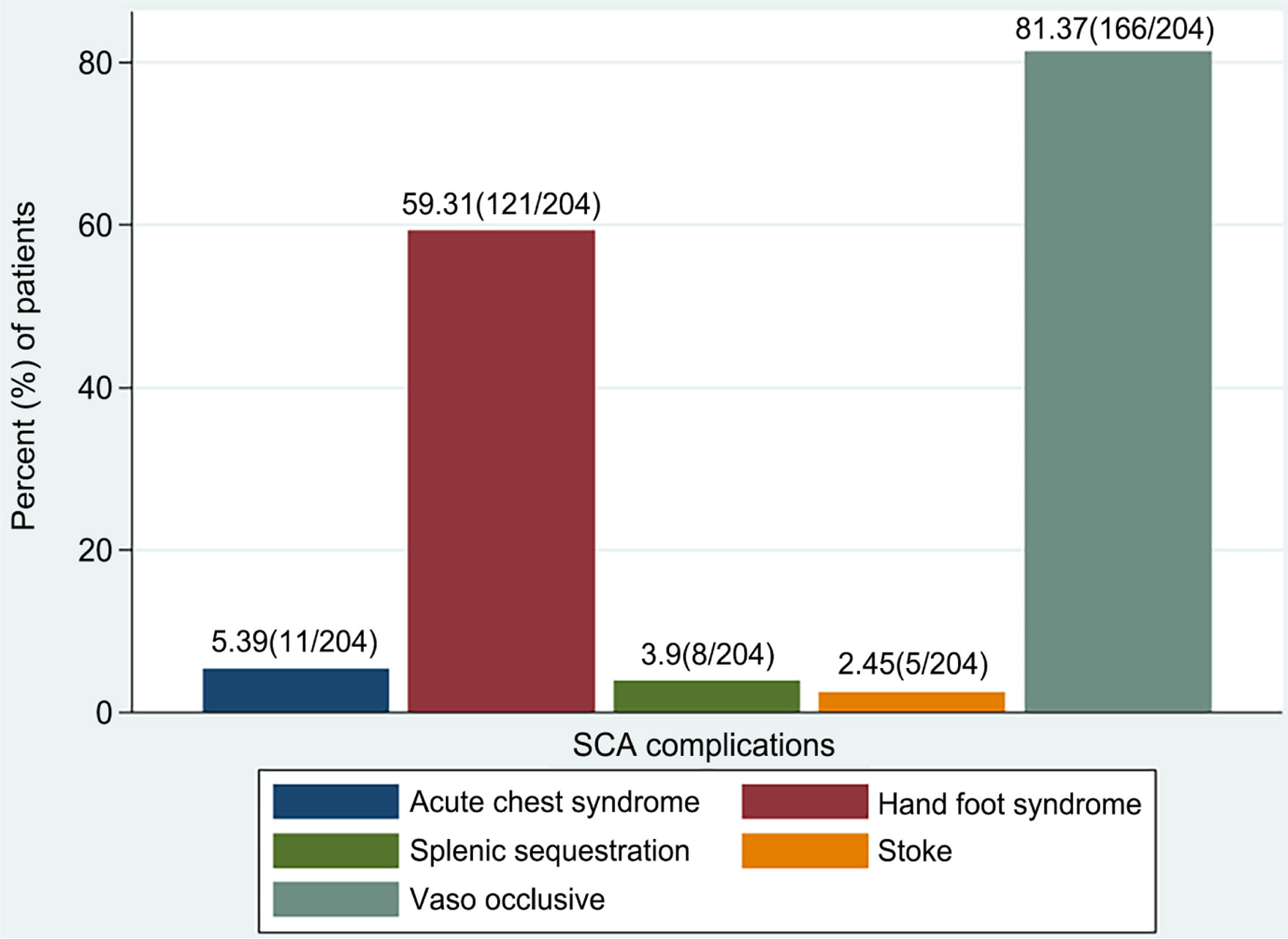
Percentage of SCA complications of 204 children with sickle cell anaemia attended in the clinic during the study period October 2016-March 2017.

The large majority of children had experienced a prior vaso-occlusive episode (166/204, 81.4%), and very few had experienced a prior stroke (5/204, 2.5%) (Figure 3).

### 3.3. Health Related Quality of Life

Of the 204 children with SCA, 163/204 (79.9%) had good HRQoL, while 41/204 (20.1%) had poor HRQoL. The mean HRQoL among 204 children with SCA was 78 (SD ± 12.45). The lowest functional domain was the school functioning with a mean score of 62.3 (SD ± 20) (Table 3).

### 3.4. Factors Associated with Poor Health Related Quality of Life

After multivariate analysis the following factors were found to be associated with poor HRQoL: age ≥ 5 years (OR = 16.2; 95% CI 4.6 – 57.5; p < 0.001), severe anaemia (OR = 4.6; 95% CI = 1.8 – 11.8; p = 0.001), severe malnutrition (OR 3; 95%CI = [1 – 9.6]; P = 0.053) and hospitalizations (OR = 3.4; 95% CI = 1.4 – 8.5; p = 0.008) (see Table 4).

## 4. Discussion

In this study, we sought to evaluate the HRQoL of children with SCA attending an outpatient clinic in Tanzania. HRQoL can be affected by local sociocultural context in addition to disease-specific factors. Globally, SCA is most prevalent in sub-Saharan Africa, and within Tanzania the Northwestern region around this clinic has the highest prevalence of the disease, [5] [6] [7] [15] with 21% of children carrying the sickle cell gene (HbAS), and more than 1% having SCA [7] [15]. We previously described the complications experienced by patients in this setting, [8] but few studies have touched on the impact that SCA has on HRQoL in paediatric populations in low-resource settings of sub-Saharan Africa. We found that 20% have a poor HRQoL, and that the median HRQoL score in this group was 78.

Few studies of HRQoL in children with SCA have been performed in sub-Saharan Africa in general or Tanzania specifically. The few studies that have attempted to measure the impact of SCA on quality of life have focused on adolescents and adult patients or caregivers of children with SCA [16]. Many studies have been conducted in high resource settings that describe HRQoL for SCA [17]. In general, the HRQoL in these other studies is worse than the HRQoL that we describe. Our findings are even more astonishing when comorbidities of the children enrolled are taken into consideration: 30.9% of the children had severe anaemia, and 12.7% of children had malnutrition. Both of these should tend to decrease their quality of life.

The higher than expected HRQoL found in our study might be related to the population that we enrolled or the suitability of the instrument that we used. The PedsQL has been validated in the paediatric SCA population [18], but it has not been validated in the sub-Saharan African context. We enrolled a younger population than some studies, but very few of them had received disease modifying therapy with hydroxyurea, so they will have encountered the same number of acute complications as children in other settings, even if they have not acquired the same degree of chronic complications [19]. Finally, younger children’s results were reported through parental proxy. It is unclear how the sociocultural context might influence parents’ answers to some of the questions on the PedsQL.

We identified several predictors of HRQoL in our study. Severe anaemia, recurrent hospitalizations, severe malnutrition, and age ≥ 5 years were all associated with a worse quality of life. In the US, Hooker [20] concluded that children who had higher haemoglobin had better HRQoL than those who had lower haemoglobin. Additionally, in a study by Brewer *et al*., SCA children who had history of blood transfusion had worse HRQoL compared to those who had not previously received a transfusion. In a low-resource setting, it is unclear how much concomitant nutritional deficiency and/or malarial infections might contribute to the severity of anaemia and overall health state, but regardless of the origin of anaemia, lower haemoglobin correlates with poor HRQoL. SCA disease modification with hydroxyurea improves the baseline haemoglobin and would likely improve the HRQoL through both improved haemoglobin concentration and fewer SCA related clinical events.

In addition, Age ≥ 5 years was found to be associated with poor HRQoL. This finding is consistent with several studies that have shown an increasing age has a negative correlation with HRQoL [10] [20] [21]. This may be attributed to an increase in severity of SCA as the patients mature with more acute events such as vaso-occlusive crisis and chronic complications.

Our study participants showed the lowest score on school functioning had the lowest scores, Similar to study done by Menezes *et al*. [22] and Dampier *et al*. [19] had lower scores in school functioning. In addition, studies done by Hooker *et al*. [20] and Dampier *et al*. [19] in America had showed lower scores in physical domain. In our study children who had history of hospitalization had lower mean score for school functioning than those who were not hospitalized. Furthermore history of recurrent hospitalizations was associated with poor HRQoL this was consistent with other studies done by Dale *et al*. [23] and Dampier *et al*. [19].

### Possible Interventions That Could Improve Quality of Life

In order to improve the quality of life to our sickle cell population, there is a great need of Incorporating HRQoL assessment tool in our guidelines so as to identify children with poor HRQoL and to intervene early by providing a comprehensive cognitive, behavioral therapy, occupational therapy, and physical therapy.

Our study limitations are due to lack of optimization of questionnaire in African children, as we had a younger population that has not acquired the same degree of poor QoL also in parental perspectives the parent proxy was not reliable.

## 5. Conclusion

This study has shown severe anaemia, older age, malnutrition and frequent hospitalizations were highly associated with poor HRQoL in a high number of children with sickle cell disease from the younger population this brings a need of providing comprehensive cognitive, behavioural therapy, occupational therapy, and physical therapy early in life.

## Figures and Tables

**Table 1.** Demographic and clinical characteristics of 204 children with sickle cell anaemia seen at Bugando medical centre clinic from October 2016 to March 2017.

Characteristic	Number (Percent) or Median (IQR)
***Male Sex***	125 (61.3)
***Median Age*** (***m****o****nths***)	72 (48 – 108)
***Age group***	
2 – 4.99 years	90 (44.1)
5.0 – 12 years	114 (55.9)
***Child education***	
Not in school	72 (35.3)
Nursery/primary school	132 (64.7)
***Relationship of caretaker***	
Aunt/Uncle/Grandparents	20 (9.8)
Parents	184 (90.2)
***Education of the caretaker***	
Illiterate	5 (2.5)
Literate	199 (98.4)
***Marital status of*** *t****he caretaker***	
Divorced	4 (2)
Married	176 (86.3)
Single	20 (9.8)
Widow/widower	4 (2)
*Median age of initial SC A presentation (months)*	6 (6 – 48)
History of hand and foot syndrome	121 (59.3)
History of VOE	166 (81.4)
History of acute chest syndrome	11 (5.4)
History of splenic sequestration	8 (3.9)
History of stroke	5 (2.5)
History of hospitalization	82 (40.1)
History of blood transfusions	134 (65.7)
History of using hydroxyurea	23 (11.3)

VOE = vaso-occlusive episode.

**Table 2.** Common clinical presentation of 204 children with sickle cell anaemia attended in the clinic during the study period October 2016-march 2017.

Sign or symptom	No. Percent (%)
**Pale**	142 (69.6)
**Jaundice**	134 (65.7)
**Oxygen saturation < 90%**	51 (25.0)
**Anaemia mild-mod anaemia**	141 (69.2)
**Severe anaemia**	63 (30.9%)
**Splenomegaly**	39 (19.0)
**Teeth malocclusion**	29 (14.2)
**Body pain**	26 (12.7)
**Tachycardia**	24 (11.8)
**Bossing of the skull**	20 (9.8)
**Hepatomegaly**	9 (4.4)
**Tachypnoea**	7 (3.4)
**Febrile illness**	6 (2.9)
**Abnormal CNS findings**	6 (2.9)
**Abnormal lungs auscultation findings**	1 (0.5)
**Leg ulcers**	1 (0.5)
***Nutritional status*** (***weight for height and BMI for age)***	
Normal	95 (46.6)
Mild wasting	45 (22.1)
Moderate wasting	38 (18.6)
Severe wasting	26 (12.7)
***Nutritional status (Height for age)***	
Normal	128 (62.8)
Mild stunting	31 (15.2)
Moderate stunting	39 (19.1)

BMI = body mass index.

**Table 3.** Health related quality of life of 204 children with sickle cell anaemia attended in the clinic during the study period October 2016-March 2017.

Domain of HRQoL	Number of patients	Mean score	Standard deviation
Mean HRQoL	204	78	12.45
Physical functioning	204	73.4	17.23
Social functioning	204	84.1	10.47
Emotional functioning	204	87.9	16
School functioning	132	62.3	20

HRQoL = health-related quality of life.

**Table 4.** Factors associated with poor health related quality of life of 204 children with sickle cell anaemia attended in the clinic during the study period October 2016-March 2017.

Factor		Quality of life		Univariate		Multivariate	
		Poor	Good	OR [95% CI]	p-value	OR [95% CI]	p-value
		n (%)	n (%)				
Sex	Female	13 (16.5)	66 (83.5)	1.0			
	Male	28 (22.4)	97 (77.6)	1.5 [0.7 – 3.0]	0.304	1.2 [0.5 – 2.7]	0.700
Age	<5 years	4 (5.3)	72 (94.7)	1.0			
	>5 years	37 (37)	91 (71.1)	7.3 [2.5 – 21.5]	<0.001	16.2 [4.6 – 57.5]	**<0.001**
Severe anaemia	No	23 (16.3)	118 (83.7)	1.0			
	Yes	18 (28.6)	45 (71.4)	2.1 [1.01 – 4.2]	0.046	4.6 [1.8 – 11.8]	**0.001**
Nutritional status	No malnutrition	18 (19)	77 (81.1)	1.0			
	Mild malnutrition	8 (17.8)	37 (82.2)	0.9 [0.4 – 2.3]	0.868		
	Mod malnutrition	6 (15.8)	32 (84.2)	0.8 [0.3 – 2.2]	0.669		
	Sev malnutrition	9 (34.6)	17 (65.4)	2.3 [0.9 – 5.9]	0.094	3 [1 – 9.6]	**0.053**
Hydroxyurea	No	33 (18.2)	148 (81.8)	1.0			
	Yes	8 (34.8)	15 (65.2)	2.4 [0.9 – 6.1]	0.068	2 [0.6 – 6.1]	0.275
Blood transfusion	No	8 (11.4)	62 (88.6)	1.0			
	Yes	33 (24.6)	101 (75.4)	2.5 [1.1 – 5.8]	0.029	1.7 [0.6 – 4.3]	0.294
Hospitalizations	No	18 (14.8)	104 (85.3)	1.0			
	Yes	23 (28.1)	59 (72)	2.3 [1.1 – 4.5]	0.022	3.4 [1.4 – 8.5]	**0.008**

## Data Availability

Data are available on reasonable request.

## Appendices

Questionnaire: English Version

### CLINICAL SPECTRUM OF CHILDREN WITH SICKLE CELL ANEMIA ATTENDING BUGANDO MEDICAL CENTER

#### SECTION 0: INSTRUCTIONS FOR THE INTERVIEWER

Obtain written consent from the patient before you fill out this questionnaire. Only use a pen when completing the questionnaire.

#### SECTION 1: SOCIAL DEMOGRAPHIC INFORMATION

**Table:**

QNo.	Questions and Instructions	Response	Code
1	Study ID Number		
2	What is the sex of the child	Male	1
	Circle the correct answer	Female	2
3	What is the age of the patient?		
4	Child’s education	Nursery school	1
		Primary school (what grade?	2
		Secondary School (what level form one-four)	3
		Position (performance in the class)	
		Number of days missed school in this term	
5	What is the ethnic background of the mother?		
6	What is the ethnic background of the father?		
7	Residence Distance to BMC (average in KM) Money spent on transport to BMC-TSHS Average amount of money spent on clinic per day Money spent on last admission		
8	What is the relationship of the caretaker to the patient?	Parent	1
		Grandparents	2
		Other (specify)	3
9	What is the level of education of the caretaker?	Never went to school	1
		Complete primary school	2
		Incomplete primary school	3
		Secondary (form one-four)	4
		Secondary (form five-six)	5
		University/college	6
10	What is the employment status of the parents/caretaker?	Peasant	1
		Teacher	2
		Carpenter	3
		Nurse	4
		Doctor	5
		Self-employed	6
		Unemployed	7
		Others (specify)	8
11	What is the Marital status of the parent/caretaker?	Married	1
		Single	2
		Divorced	3
		Others (specify)	4
12	Is there a sibling or family member with SCD?	Yes Confirmed	1
		Not Confirmed	
		No	2
13	Is there a family member who has done Sickling test?	Positive	1
		Negative	2

#### SECTION 2: DISEASE COURSE

**Table:**

QNo.	Questions and Instructions	Response	Code
1	At what age did the child first present with signs and symptoms of SCA? Which one?		
2	Has the child ever received blood transfusion?	Yes…total number of BT When was the last BT?	
		No
3	What was the age of the first blood transfusion?		
4	Total number of admissions since diagnosis SCA, of days/weeks hospitalized in the last admission?		
5	Total number of admissions in the past six months		

#### SECTION 3: CURRENT HISTORY

**Table:**

QNo.	Questions and Instructions	Response	Code
1	Is the child well today?	Yes	1
		No	2
2	Does the child have febrile illness?	Yes	1
		No	2
3	Does the child have cough?	Yes	1
		No	2
4	Does the child have pain?	Yes	1
		chest pain abdominal pain back pain Joint pain headache other-(specify)	
		No	2
5	Does the child have joint swelling or toes or fingers swelling? or swelling anywhere	No	1
		Yes	2
		If yes (specify)	
6	Does the child have yellowish discoloration of the eyes?	No	1
		Yes	2
7	Does the child have hx of nasal bleeding?	Yes	1
		No	2
8	Does the child have hx of leg ulcers?	Yes	1
		No	2

#### SECTION 4: PHYSICAL EXAMINATION

**Table:**

QNo.	Question and Instructions	Response	Codes
1	Pallor	Yes	1
		No	2
2	Jaundice	Yes	1
		No	2
3	Pulse rate	Normal	1
		Bradycardia	2
		Tachycardia	3
4	Respiratory rate	Normal	1
		Abnormal	2
5	oxygen saturation	>90% in room air	1
		<90% in room air	2
6	Weight		
7	Height		
8	Weight for height	No malnutrition	0
		Mild malnutrition	1
		Moderate malnutrition	2
		Severe malnutrition	3
9	Height for Age	No stunting	1
		Mild stunting	2
		Moderate stunting	3
		Severe stunting	4
10	BMI for Age	No malnutrition	1
		Mild malnutrition	2
		Moderate malnutrition	3
		Severe malnutrition	4
11	Tanner stage	stage 1	1
		stage 2	2
		stage 3	3
		stage 4	4
		stage 5	5
12	Malocclusion	Yes	1
		No	2
13	Bossing of the skull	Yes	1
		No	2
14	Leg ulcers	Yes	1
		No	2
15	Hepatomegaly	Yes	1
		No	2
		Size of the liver	
16	Splenomegaly	Yes	1
		No	2
		Size of the spleen	
17	Abnormal CNS findings	Yes	1
		No	2
		If yes (specify)	
18	Abnormal auscultations findings	Yes	1
		No	2
19	Any medical diagnosis found on evaluation today (mention)	Yes	1
		No	2
		If yes (specify)	

#### SECTION 5: SCA RELATED COMPLICATIONS PAST AND CURRENT

**Table:**

QNo.	Question and Instructions	Response	Codes
1	Acute chest syndrome	None	1
		Once	2
		more than once	3
2	Hand and foot syndrome	None	1
		Once	2
		more than once	3
3	Splenic sequestration	None	1
		Once	2
		more than once	3
4	Stroke	None	1
		Once	2
		More than once	3
5	VOC (specify)-Acute abdomen -Bone and Joint pain	None	1
		Once	2
		more than once	3
6	Hyperhemolysis	None	1
		Once	2
		more than once	3
7	Aplastic Anemia	None	1
		Once	2
		more than once	3

#### SECTION 6: CURRENT MEDICATION AND LABORATORY RESULTS

**Table:**

QNO	Questions and Instructions	Response	Codes
1	Is the child on Folic acid?	Yes	1
		No	2
2	Is the child on Pen V?	Yes	1
		No	2
3	Is the child on Hydroxyurea? When did she/he start?	Yes	1
		No	2
4	Other medications? Herbal? Iron supplements? any alternative treatment? (specify)	Yes (specify)	1
		No	2
5	Any laboratory investigations required today(not part of this study)		

#### SECTION 7: LABORATORY RESULTS

**Table:**

FBP RESULTS	WBC
	HB
	HCT
	PLT
	MCV
	MCHC
	RETICULOCYTE COUNT
	LYMPHOCYTES
	NEUTROPHILS
	EOSINOPHILS
	MONOCYTES
